# Whole-Genome Sequence of Fluoroquinolone-Resistant Escherichia coli HUE1, Isolated in Hokkaido, Japan

**DOI:** 10.1128/MRA.01135-20

**Published:** 2020-11-12

**Authors:** Montgomery Munby, Jumpei Fujiki, Kotaro Aoki, Chika Kawaguchi, Keisuke Nakamura, Tomohiro Nakamura, Michihito Sasaki, Toyotaka Sato, Masaru Usui, Hirofumi Sawa, Shin-ichi Yokota, Yutaka Tamura, Hidetomo Iwano

**Affiliations:** a Laboratory of Veterinary Biochemistry, Rakuno Gakuen University School of Veterinary Medicine, Ebetsu, Japan; b Department of Microbiology and Infectious Diseases, Toho University School of Medicine, Tokyo, Japan; c Division of Molecular Pathobiology, Research Center for Zoonosis Control, Hokkaido University, Sapporo, Japan; d Department of Microbiology, Sapporo Medical University School of Medicine, Sapporo, Japan; e Laboratory of Food Microbiology and Food Safety, Rakuno Gakuen University School of Veterinary Medicine, Ebetsu, Japan; f International Collaboration Unit, Research Center for Zoonosis Control, Hokkaido University, Sapporo, Japan; g Center for Veterinary Drug Development, Rakuno Gakuen University, Ebetsu, Japan; University of Maryland School of Medicine

## Abstract

We report the complete genome sequence of Escherichia coli strain HUE1, isolated from the urinary catheter of a female patient, showing fluoroquinolone resistance without quinolone resistance-determining region mutations. To facilitate the exploration of the molecular characteristics of HUE1, the whole genome was sequenced using long- and short-read platforms.

## ANNOUNCEMENT

Escherichia coli strain HUE1 was isolated in 2008 from the urinary catheter of a 77-year-old female patient at Hokkaido University Hospital in Japan (1). The specimen was cultured on sheep blood agar and MacConkey agar medium under aerobic incubation. The isolate was identified with MicroScan WalkAway (Beckman Coulter, Brea, CA, USA). The strain exhibited antimicrobial resistance (AMR) against fluoroquinolones (1, 2).

Mutations in the quinolone resistance-determining region (QRDR) of the chromosomally located genes (*gyrA*, *gyrB*, *parC*, and *parE*) was believed to be required for exceeding breakpoints of fluoroquinolone MICs and the acquisition of the fluoroquinolone-resistant phenotype (3–5). Plasmid-mediated quinolone resistance (PMQR) genes [such as *qnr*, *oqxAB*, and *aac(6′)-Ib-cr*] are also involved in fluoroquinolone resistance in E. coli (6, 7). Although no mutations were found in the QRDR of HUE1, the *acrAB* and *tolC* multidrug efflux pump gene-deficient HUE1 mutant exhibited great reductions of fluoroquinolone MICs (1, 2), indicating that *tolC*-mediated efflux systems are critical for the acquisition of fluoroquinolone resistance. Here, we sequenced the whole HUE1 genome and report the molecular characteristics of this unique E. coli isolate.

Genomic DNA was extracted from HUE1 grown in overnight culture at 37°C in LB medium using phenol and chloroform (8) and was purified by a QIAamp DNA minikit column (Qiagen, Hilden, Germany). The DNA concentration was determined using a Qubit fluorometer (Invitrogen, Carlsbad, CA, USA). A short-read sequencing library was prepared using a Nextera XT DNA library preparation kit (Illumina, San Diego, CA, USA) according to the manufacturer’s protocol. The whole genome was then 300-bp paired-end sequenced on the MiSeq platform (Illumina). The resulting 5,126,454 reads were trimmed of adaptors and low-quality bases (Q score, <20), and short reads (<36 bp) were removed using Trimmomatic v0.39 (9), resulting in a total of 4,662,794 reads (220× coverage). A long-read sequencing library was prepared using a rapid barcoding kit (Oxford Nanopore Technologies, Oxford, UK) according to the manufacturer’s protocol. The resulting sample was loaded onto an R9.4 flow cell (Oxford Nanopore Technologies) and sequenced using MinION sequencing (Oxford Nanopore Technologies). The obtained 786,618 reads were demultiplexed using Porechop v0.2.4 (https://github.com/rrwick/Porechop), and then the reads were adaptor trimmed and quality filtered using Albacore v2.3.4 and Nanofilt (Q score, <8; minimum length, 1,000 bp) (10, 11). The reads were randomly subsampled using seqtk v1.3.-r106 (https://github.com/lh3/seqtk) down to 100,000 reads (*N*_50_, 11,359 bp; 149× coverage). Hybrid *de novo* assembly was performed using Unicycler v0.4.8 beta (12), which revealed six circular contigs with a total length of 4,742,352 bp. For each contig, the Unicycler pipeline automatically detected and trimmed overlaps, revealing all six of the contigs to be circular, and then it rotated the chromosome and plasmids to begin with *dnaA* and *repA*. Finally, the assembled sequences were annotated using DFAST v1.1.0 with standard settings (13).

The genome of HUE1 consists of one chromosome and five plasmids with a total of 4,462 coding sequences, 22 rRNAs, and 88 tRNAs (Table 1). Plasmid replicon types were identified using PlasmidFinder 2.1 (14). ResFinder 4.0 analysis (15) detected 1 AMR gene in the chromosome and 11 AMR genes in the plasmids that might be related to the multidrug resistance of HUE1. No mutations were detected in the HUE1 QRDR. These results suggest that the mechanism underlying HUE1 fluoroquinolone resistance without QRDR mutations involves PMQR genes and efflux transporters, as previously reported (1, 2).

**TABLE 1:** Characteristics of the E. coli HUE1 genome

Contig	Component	Name	Length (bp)	G+C (%)	Plasmid replicon type^*a*^	AMR gene(s)
1	Chromosome	HUE1 chromosome	4,557,972	50.93		*mdf*(A)
2	Plasmid	pHFQ1	120,829	49.52	p0111	*aac(5)–Iia*, *aadA5*, *floR*, *oqxA*, *oqxB*, *drfA17*, *sul1*, *sul2*
3	Plasmid	pHFQ2	48,997	43.42	IncX1	*qnrS1*, *bla*_TEM-1B_
4	Plasmid	pHFQ3	5,501	40.36	NI	
5	Plasmid	pHFQ4	5,498	63.33	NI	*tet*(A)
6	Plasmid	pHFQ5	3,555	45.65	NI	

a NI, not identified in the PlasmidFinder analysis.

### Data availability.

The complete genome sequence of E. coli HUE1 was deposited in DDBJ/ENA/GenBank under accession numbers AP023427, AP023428, AP023429, AP023430, AP023431, and AP023432. Illumina and MinION sequence reads for the strain were deposited in the Sequence Read Archive (SRA) database under accession numbers DRR241416 and DRR241417, respectively.

## References

[1] Sato T, Yokota S, Uchida I, Okubo T, Ishihara K, Fujii N, Tamura Y 2011 A fluoroquinolone-resistant Escherichia coli clinical isolate without quinolone resistance-determining region mutations found in Japan. Antimicrob Agents Chemother 55:3964–3965. doi:10.1128/AAC.00532-11. 21670180 PMC3147648

[2] Sato T, Yokota S, Uchida I, Okubo T, Usui M, Kusumoto M, Akiba M, Fujii N, Tamura Y 2013 Fluoroquinolone resistance mechanisms in an Escherichia coli isolate, HUE1, without quinolone resistance-determining region mutations. Front Microbiol 4:125. doi:10.3389/fmicb.2013.00125. 23745120 PMC3662882

[3] Heisig P 1996 Genetic evidence for a role of parC mutations in development of high-level fluoroquinolone resistance in Escherichia coli. Antimicrob Agents Chemother 40:879–885. doi:10.1128/AAC.40.4.879. 8849244 PMC163223

[4] Conrad S, Oethinger M, Kaifel K, Klotz G, Marre R, Kern WV 1996 gyrA mutations in high-level fluoroquinolone-resistant clinical isolates of Escherichia coli. J Antimicrob Chemother 38:443–455. doi:10.1093/jac/38.3.443. 8889719

[5] Breines DM, Ouabdesselam S, Ng EY, Tankovic J, Shah S, Soussy CJ, Hooper DC 1997 Quinolone resistance locus nfxD of Escherichia coli is a mutant allele of the parE gene encoding a subunit of topoisomerase IV. Antimicrob Agents Chemother 41:175–179. doi:10.1128/AAC.41.1.175. 8980775 PMC163680

[6] Jacoby GA 2005 Mechanisms of resistance to quinolones. Clin Infect Dis 41(Suppl 2):S120–S126. doi:10.1086/428052. 15942878

[7] Hansen LH, Jensen LB, Sorensen HI, Sorensen SJ 2007 Substrate specificity of the OqxAB multidrug resistance pump in Escherichia coli and selected enteric bacteria. J Antimicrob Chemother 60:145–147. doi:10.1093/jac/dkm167. 17526501

[8] Sambrook J, Russell DW 2006 Purification of nucleic acids by extraction with phenol:chloroform. CSH Protoc 2006:pdb.prot4455. doi:10.1101/pdb.prot4455. 22485786

[9] Bolger AM, Lohse M, Usadel B 2014 Trimmomatic: a flexible trimmer for Illumina sequence data. Bioinformatics 30:2114–2120. doi:10.1093/bioinformatics/btu170. 24695404 PMC4103590

[10] De Coster W, D'Hert S, Schultz DT, Cruts M, Van Broeckhoven C 2018 NanoPack: visualizing and processing long-read sequencing data. Bioinformatics 34:2666–2669. doi:10.1093/bioinformatics/bty149. 29547981 PMC6061794

[11] Wick RR, Judd LM, Gorrie CL, Holt KE 2017 Completing bacterial genome assemblies with multiplex MinION sequencing. Microb Genom 3:e000132. doi:10.1099/mgen.0.000132. 29177090 PMC5695209

[12] Wick RR, Judd LM, Gorrie CL, Holt KE 2017 Unicycler: resolving bacterial genome assemblies from short and long sequencing reads. PLoS Comput Biol 13:e1005595. doi:10.1371/journal.pcbi.1005595. 28594827 PMC5481147

[13] Tanizawa Y, Fujisawa T, Nakamura Y 2018 DFAST: a flexible prokaryotic genome annotation pipeline for faster genome publication. Bioinformatics 34:1037–1039. doi:10.1093/bioinformatics/btx713. 29106469 PMC5860143

[14] Carattoli A, Zankari E, Garcia-Fernandez A, Voldby Larsen M, Lund O, Villa L, Moller Aarestrup F, Hasman H 2014 In silico detection and typing of plasmids using PlasmidFinder and plasmid multilocus sequence typing. Antimicrob Agents Chemother 58:3895–3903. doi:10.1128/AAC.02412-14. 24777092 PMC4068535

[15] Bortolaia V, Kaas RS, Ruppe E, Roberts MC, Schwarz S, Cattoir V, Philippon A, Allesoe RL, Rebelo AR, Florensa AF, Fagelhauer L, Chakraborty T, Neumann B, Werner G, Bender JK, Stingl K, Nguyen M, Coppens J, Xavier BB, Malhotra-Kumar S, Westh H, Pinholt M, Anjum MF, Duggett NA, Kempf I, Nykasenoja S, Olkkola S, Wieczorek K, Amaro A, Clemente L, Mossong J, Losch S, Ragimbeau C, Lund O, Aarestrup FM 11 8 2020 ResFinder 4.0 for predictions of phenotypes from genotypes. J Antimicrob Chemother doi:10.1093/jac/dkaa345. PMC7662176 32780112

